# Rates of cortical thinning in Alzheimer’s disease signature regions associate with vascular burden but not with β-amyloid status in cognitively normal adults at age 70

**DOI:** 10.1136/jnnp-2023-332067

**Published:** 2024-01-10

**Authors:** Sarah E Keuss, William Coath, David M Cash, Josephine Barnes, Jennifer M Nicholas, Christopher A Lane, Thomas D Parker, Ashvini Keshavan, Sarah M Buchanan, Aaron Z Wagen, Mathew Storey, Matthew Harris, Kirsty Lu, Sarah-Naomi James, Rebecca Street, Ian B Malone, Carole H Sudre, David L Thomas, John C Dickson, Frederik Barkhof, Heidi Murray-Smith, Andrew Wong, Marcus Richards, Nick C Fox, Jonathan M Schott

**Affiliations:** 1 Dementia Research Centre, UCL Queen Square Institute of Neurology, University College London, London, UK; 2 Dementia Research Institute, University College London, London, UK; 3 Department of Medical Statistics, London School of Hygiene & Tropical Medicine, London, UK; 4 Department of Brain Sciences, Imperial College London, London, UK; 5 Neurodegeneration Biology Laboratory, The Francis Crick Institute, London, UK; 6 Genetics and Genomic Medicine, Great Ormond Street Institute of Child Health, University College London, London, UK; 7 MRC Unit for Lifelong Health and Ageing at UCL, University College London, London, UK; 8 School of Biomedical Engineering & Imaging Sciences, King's College London, London, UK; 9 Centre for Medical Imaging Computing, University College London, London, UK; 10 Department of Brain Repair and Neurorehabilitation, UCL Queen Square Institute of Neurology, University College London, London, UK; 11 Institute of Nuclear Medicine, University College London Hospitals NHS Foundation Trust, London, UK; 12 Department of Radiology and Nuclear Medicine, Amsterdam University Medical Centre, Vrije Universiteit Amsterdam, Amsterdam, Netherlands

**Keywords:** CEREBROVASCULAR DISEASE, AMYLOID, ALZHEIMER'S DISEASE

## Abstract

**Background:**

Consistent patterns of reduced cortical thickness have been identified in early Alzheimer’s disease (AD). However, the pathological factors that influence rates of cortical thinning within these AD signature regions remain unclear.

**Methods:**

Participants were from the Insight 46 substudy of the MRC National Survey of Health and Development (NSHD; 1946 British birth cohort), a prospective longitudinal cohort study. Linear regression was used to examine associations of baseline cerebral β-amyloid (Aβ) deposition, measured using florbetapir positron emission tomography, and baseline white matter hyperintensity volume (WMHV) on MRI, a marker of cerebral small vessel disease, with subsequent longitudinal changes in AD signature cortical thickness quantified from baseline and repeat MRI (mean [SD] interval 2.4 [0.2] years).

**Results:**

In a population-based sample of 337 cognitively normal older white adults (mean [SD] age at baseline 70.5 [0.6] years; 48.1% female), higher global WMHV at baseline related to faster subsequent rates of cortical thinning in both AD signature regions (~0.15%/year faster per 10 mL additional WMHV), whereas baseline Aβ status did not. Among Aβ positive participants (n=56), there was some evidence that greater global Aβ standardised uptake value ratio at baseline related to faster cortical thinning in the AD signature Mayo region, but this did not reach statistical significance (p=0.08).

**Conclusions:**

Cortical thinning within AD signature regions may develop via cerebrovascular pathways. Perhaps reflecting the age of the cohort and relatively low prevalence of Aβ-positivity, robust Aβ-related differences were not detected. Longitudinal follow-up incorporating additional biomarkers will allow assessment of how these relationships evolve closer to expected dementia onset.

## Introduction

Previous MRI studies have identified consistent patterns of decreased cortical thickness in early Alzheimer’s disease (AD)—termed AD signatures—which predict cognitive decline and AD dementia in cognitively normal (CN) older adults.^1–4^ Similar findings have also been detected longitudinally in presymptomatic autosomal dominant AD.^5^

The relationship between β-amyloid (Aβ) deposition, one of the neuropathological hallmarks of AD and cortical thickness is unclear. Findings from MRI studies have been mixed, with some studies reporting Aβ-related reductions in cortical thickness,^3 6^ while a previous cross-sectional analysis from our group did not detect significant differences,^7^ and some researchers have observed Aβ-associated increases in cortical thickness.^8^ This has led to suggestions that the relationship may be non-linear or perhaps mediated via or interactive with tau pathology or other disease processes.^9^ Alternatively, discrepancies between studies might relate to difficulties accounting for heterogeneity between individuals, either reflecting premorbid differences in brain structure—an issue in cross-sectional studies—or the effects of age and other pathologies which often coexist in later life.

To investigate this further, this study examines associations of baseline cerebral Aβ deposition, measured using florbetapir positron emission tomography (PET), and baseline white matter hyperintensity volume (WMHV) on MRI, a marker of cerebral small vessel disease (CSVD), with subsequent longitudinal changes in AD signature cortical thickness quantified from MRI in CN older adults of almost identical age.

## Methods

Participants were scanned on a single Biograph mMR 3T PET/MRI (Siemens Healthcare) at two time points as part of the Insight 46 substudy of the MRC National Survey of Health and Development (NSHD; the 1946 British birth cohort).^10^

Baseline Aβ PET data obtained postinjection of 370 MBq 18F florbetapir were processed with pseudo-CT attenuation correction.^11^ Global standardised uptake value ratios (SUVRs) were generated using a cortical region of interest, based on a previously defined composite,^12^ and an eroded subcortical white matter reference region. A gaussian mixture model was applied to global SUVRs and the 99th percentile of the lower gaussian was taken as the cut-point for Aβ positivity (0.6104).

Baseline global WMHV was measured from distortion-corrected and bias-corrected T1 and fluid-attenuated inversion recovery MRI data using an unsupervised automated algorithm, Bayesian Model Selection, as described elsewhere.^13^

**Figure 1:**
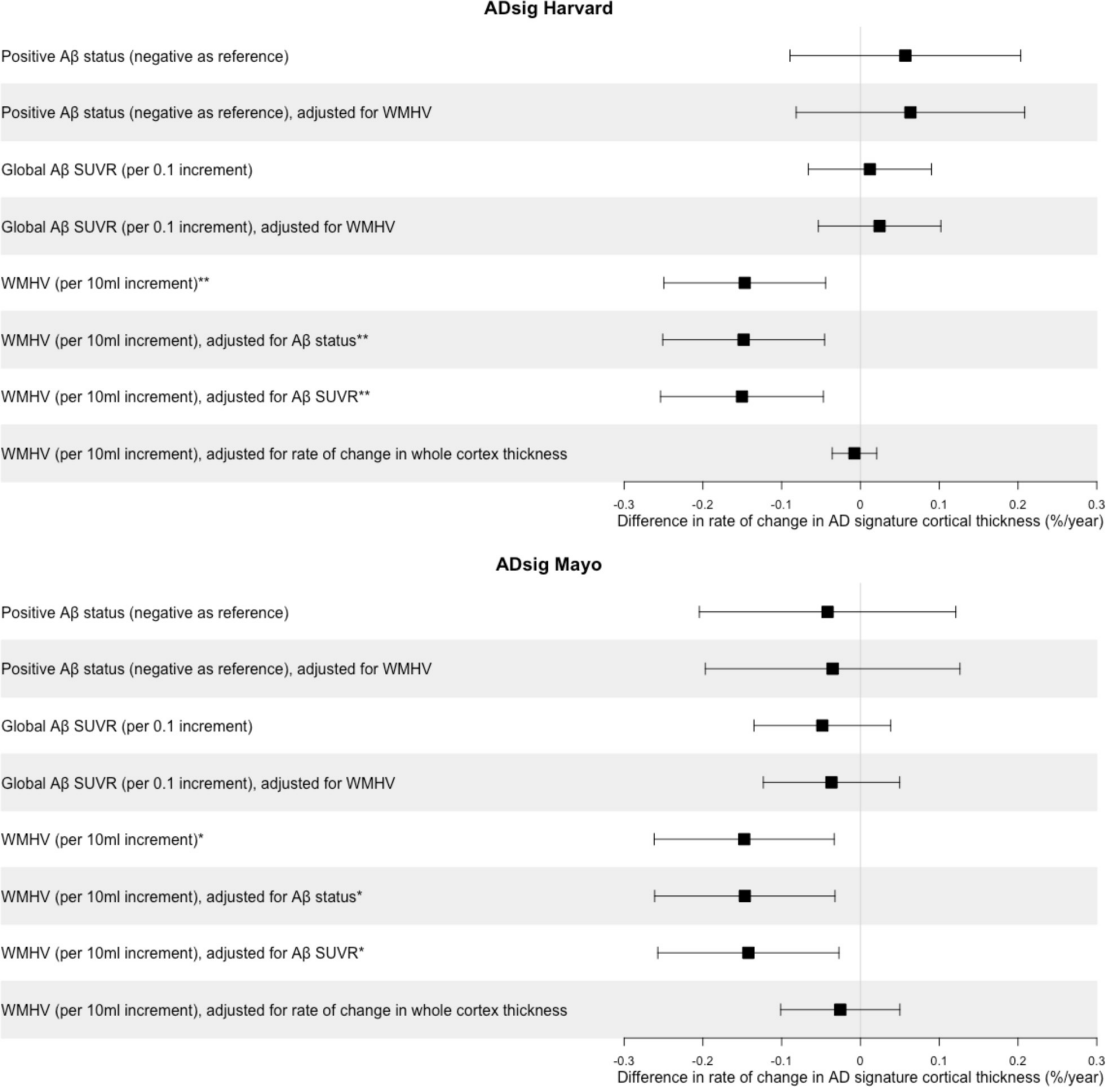
Associations of baseline Aβ deposition and baseline WMHV with subsequent rates of change in cortical thickness in Alzheimer’s disease (AD) signature regions in cognitively normal participants. Coefficients and 95% CIs are presented from linear regression models, adjusted for sex and age at baseline scan. *p≤0.05; **p≤0.01. ADsig Harvard consisted of entorhinal, inferior temporal, parahippocampal, temporal pole, precuneus, supramarginal, superior and inferior parietal, superior frontal, pars opercularis, pars triangularis and pars orbitalis areas.^3^ ADsig Mayo was composed of middle temporal, inferior temporal, entorhinal and fusiform areas.^4^ Aβ, β-amyloid; SUVR, standardised uptake value ratio; WMHV, white matter hyperintensity volume.

Cortical thickness was estimated at each time point using Freesurfer V.7.1.0 (https://surfer.nmr.mgh.harvard.edu/). Distortion-corrected T1 MRI underwent cross-sectional processing within Freesurfer, before being processed through the longitudinal stream.^14^ To form the AD signatures (ADsig Harvard and Mayo; see figure 1 for region descriptions), Desikan-Killian atlas labels were merged and single annotation files were created.^15^ Surface area-weighted averages of extracted left and right hemisphere AD signature cortical thickness values were then calculated.

Statistical analyses were performed in STATA V.17. Overall, 356 of 502 participants had high-quality longitudinal MRI data, of whom those with dementia (n=2), mild cognitive impairment (n=4), other confounding brain disorders (n=4) or missing Aβ or WMHV data (n=9) at baseline were excluded.^16^

Differences in baseline characteristics by Aβ status were assessed using t-tests, Wilcoxon rank-sum tests or χ^2^ tests, as appropriate.

Associations of baseline Aβ (status or global SUVR) and baseline global WMHV with subsequent changes in AD signature cortical thickness were tested using linear regression models, similar to those previously described.^16^ Effects of Aβ and WMHV were assessed in separate models and then as predictors in a single model, with adjustment for sex and baseline age. WMHV was not corrected for total intracranial volume since this did not alter the results (online supplemental etable 1).

10.1136/jnnp-2023-332067.supp1 Supplementary data

Model assumptions were checked by examination of residual plots. Non-linear relationships were assessed by inspection of residual versus predictor plots and were formally tested by adding quadratic terms to models.

## Results

A total of 337 CN participants (mean [SD] age 70.5 [0.6] years; 48.1% female) had complete imaging data (mean [SD] scan interval 2.4 [0.2] years). There were significantly more APOE ε4 carriers among Aβ positive than Aβ negative participants (60.7% vs 22.6%; p<0.001), but age, sex and other baseline characteristics did not differ significantly by Aβ status (table 1).

**Table 1:** Participant characteristics

Characteristic		All participants (n=337)	Aβ positive (n=56)	Aβ negative (n=281)
Age at baseline visit, years, mean (SD)		70.5 (0.6)	70.5 (0.6)	70.5 (0.6)
Sex, % female		48.1	42.9	49.1
Childhood cognition, z-score, mean (SD)		0.41 (0.72)	0.34 (0.71)	0.43 (0.73)
Education level	% none	15.4	17.9	15.0
	% O-level or equivalent or vocational	32.3	41.1	30.6
	% A-level or equivalent or higher	52.2	41.1	54.5
Socioeconomic position at age 53, % manual occupation		15.1	16.1	15.0
APOE ε4 status, % carrier*		29.0 (n=335)†	60.7	22.6 (n=279)†
Global WMHV at baseline, mL, median (IQR)		2.7 (1.5–6.1)	3.3 (1.8–6.2)	2.6 (1.5–6.1)
PACC at baseline, z-score, mean (SD)		0.05 (0.67)	−0.04 (0.69)	0.07 (0.66)

*Significant difference detected between Aβ positive and negative groups (p≤0.001).

†Number of participants with available data if below maximum possible.

APOE, apolipoprotein E; Aβ, β-amyloid; PACC, preclinical Alzheimer’s cognitive composite; WMHV, white matter hyperintensity volume.

There were no significant relationships between baseline Aβ (status or global SUVR) and subsequent rates of change in cortical thickness in either AD signature region, whereas higher global WMHV at baseline associated with significantly faster subsequent rates of cortical thinning in both AD signature regions: 0.15%/year faster per 10 mL additional WMHV (figure 1, online supplemental etable 1). There was no material difference in the results when the effects of Aβ and WMHV were assessed as predictors in a single model; however, effects of WMHV were attenuated and non-significant after adjustment for rate of whole cortex thickness change (figure 1, online supplemental etable 1). There were no interactions between Aβ and WMHV or between sex and Aβ or WMHV, and no non-linear relationships (p>0.1, all tests).

In post hoc analyses, effects of regional (lobar) Aβ SUVR were virtually identical to each other (online supplemental etable 2). There was some evidence that, among Aβ positive participants, higher global SUVR at baseline related to faster subsequent cortical thinning in the ADsig Mayo region, though it did not reach statistical significance (p=0.08), and a similar relationship was not detected with the ADsig Harvard region (online supplemental etable 3). In a vertex-wise analysis, rates of change in cortical thickness did not differ significantly by baseline Aβ status in any brain region after cluster-wise correction for multiple comparisons (1000 permutations; cluster-forming threshold p<0.05).

## Discussion

In CN adults ~70 years old, higher baseline WMHV—a marker of CSVD—related to significantly faster subsequent rates of cortical thinning in AD signature regions, whereas baseline Aβ status did not.

The association with WMHV does not necessarily mean that CSVD has a direct role in AD pathogenesis—indeed, effects were reduced to almost zero and non-significant after adjustment for rate of whole cortex thickness change, implying that they were not disproportionate to global changes—but it may reflect that CSVD contributes to cortical thinning in later life including within regions known to be vulnerable in AD. Thus, interventions aimed at reducing development of CSVD in later life may help to slow neurodegeneration in these areas, potentially delaying or preventing progression to dementia. Moreover, studies using AD signature cortical thickness as a biomarker in AD should consider possible effects of CSVD.

Notably, rates of cortical thinning did not differ by Aβ status, either within AD signature regions or elsewhere in the brain. While there was some evidence, among Aβ positive participants, that higher global Aβ SUVR at baseline related to faster subsequent cortical thinning in the ADsig Mayo region, this was not statistically significant. Insight 46 is a relatively young cohort, and rates of Aβ-positivity (~17%) in the current sample, while broadly within those expected for age, are perhaps slightly lower than in some studies, likely reflecting that the cohort is population based.^17^ This might explain why some studies of CN adults—often with older age ranges or greater rates of Aβ-positivity—have detected significant Aβ-related cortical thinning,^3 6^ whereas Insight 46 analyses—both here and in a previous cross-sectional study^7^—have not observed robust differences.

Another potential issue is that there may be apparent ‘thickening’ of the cortex in early AD, perhaps related to a transient inflammatory response to Aβ. Evidence supporting this hypothesis is largely based on cross-sectional studies with small subject numbers and has not been widely replicated.^8 18^ However, if this were the case, effects of Aβ in opposite directions may cancel each other out when assessed at a group level, making it difficult to detect a relationship in early AD.

A further consideration is whether the absence of a significant relationship may be technique related. In the same sample, we previously reported Aβ-related differences in rates of global and hippocampal volume loss measured using the boundary shift integral (BSI).^16^ While comparison with this study is difficult due to the different regions assessed, this might reflect that the BSI is more precise, providing a direct rather than indirect measure of change, or that cortical thickness is computationally more difficult to quantify than volume. Indeed, whole brain volume change measured using the BSI was much less variable (SD/mean ratio ~1/3) than whole cortical thickness change measured using Freesurfer.

Strengths of this study include that participants were scanned on a single PET/MRI scanner at an almost identical age. A limitation is the absence of tau PET data. Previous studies have detected interactions between Aβ and tau, whereby Aβ-positivity associated with increased cortical thickness in tau negative individuals but reduced cortical thickness in tau positive individuals.^9 18^ Tau has also been suggested as a cause of WMH, perhaps via Wallerian degeneration.^19^ Other limitations include that there was insufficient power to assess the impact of other CSVD features (eg, lacunes or microbleeds) due to their low frequency in this sample^20^; and that Aβ PET may reflect both Aβ found in AD plaques and that in cerebral amyloid angiopathy,^21^ which may have confounded the results.

In conclusion, the findings in this study add to current understanding of the factors that might influence rates of change in AD signature cortical thickness in CN older adults, as well as highlighting important avenues for further research.

## Data Availability

Data are available on reasonable request. Further details can be found at http://www.nshd.mrc.ac.uk/data.
