# Supplementary material for: Rates of cortical thinning in Alzheimer’s disease signature regions associate with vascular burden but not with β-amyloid status in cognitively normal adults at age 70
Source: J Neurol Neurosurg Psychiatry. 2024 Jan 10;95(8):748–52. doi: 10.1136/jnnp-2023-332067 (PMC11287522; doi:10.1136/jnnp-2023-332067)
Supplement: Supplementary data [file jnnp-2023-332067supp001.pdf]

Supplementary eTable 1. Associations of baseline β-amyloid deposition and baseline white matter hyperintensity volume with subsequent rates of change in cortical thickness in Alzheimer's disease signature regions in cognitively normal participants.

**Predictor of interest**	**Difference in rate of change in cortical thickness in %/year (95% CI)**	
	**ADsig Harvard**	**ADsig Mayo**
Aβ status (positive versus negative)	0.06 (-0.09, 0.20)	-0.04 (-0.20, 0.12)
Aβ status (positive versus negative), adjusted for WMHV	0.06 (-0.08, 0.21)	-0.04 (-0.20, 0.13)
Global Aβ SUVR (per 0.1 increment)	0.01 (-0.07, 0.09)	-0.05 (-0.14, 0.04)
Global Aβ SUVR (per 0.1 increment), adjusted for WMHV	0.02 (-0.05, 0.10)	-0.04 (-0.12, 0.05)
WMHV (per 10mL increment)	-0.15 (-0.25, -0.04) **	-0.15 (-0.26, -0.03) *
WMHV (per 10mL increment), adjusted for TIV	-0.15 (-0.25, -0.04) **	-0.14 (-0.26, -0.03) *
WMHV (per 10mL increment), adjusted for Aβ status	-0.15 (-0.25, -0.05) **	-0.15 (-0.26, -0.03) *
WMHV (per 10mL increment), adjusted for Aβ SUVR	-0.15 (-0.25, -0.05) **	-0.14 (-0.26, -0.03) *
WMHV (per 10mL increment), adjusted for rate of change in whole cortex thickness	-0.01 (-0.04, 0.02)	-0.03 (-0.10, 0.05)

Coefficients and 95% confidence intervals are from linear regression models adjusted for sex and age at baseline scan. Aβ = β-amyloid; SUVR = standardised uptake value ratio; WMHV = white matter hyperintensity volume; TIV = total intracranial volume. * $p \leq 0.05$; ** $p \leq 0.01$. ADsig Harvard consisted of entorhinal, inferior temporal, parahippocampal, temporal pole, precuneus, supramarginal, superior and inferior parietal, superior frontal, pars opercularis, pars triangularis and pars orbitalis areas.[1] ADsig Mayo was comprised of middle temporal, inferior temporal, entorhinal and fusiform areas.[2]

Supplementary eTable 2. Associations of baseline regional (lobar) β-amyloid standardised uptake value ratios with subsequent rates of change in cortical thickness in Alzheimer's disease signature regions in cognitively normal participants.

SUVR region	Difference in rate of change in cortical thickness in %/year per 0.1 increment in baseline Aβ SUVR (95% CI)	
	**ADsig Harvard**	**ADsig Mayo**
Frontal	0.00 (-0.08, 0.09)	-0.07 (-0.16, 0.02)
Parietal	-0.00 (-0.09, 0.08)	-0.07 (-0.17, 0.02)
Temporal	0.00 (-0.09, 0.10)	-0.06 (-0.16, 0.05)
Occipital	-0.01 (-0.10, 0.08)	-0.06 (-0.16 0.03)

Note that regional SUVR data was available for 316 out of 337 participants. Coefficients and 95% confidence intervals are from linear regression models adjusted for sex and age at baseline scan. Aβ = β-amyloid; SUVR = standardised uptake value ratio. ADsig Harvard consisted of entorhinal, inferior temporal, parahippocampal, temporal pole, precuneus, supramarginal, superior and inferior parietal, superior frontal, pars opercularis, pars triangularis and pars orbitalis areas.[1] ADsig Mayo was comprised of middle temporal, inferior temporal, entorhinal and fusiform areas.[2]

Supplementary eTable 3. Associations of baseline global β-amyloid standardised uptake value ratios with subsequent rates of change in cortical thickness in Alzheimer's disease signature regions in cognitively normal participants, allowing for differing slopes in β-amyloid positive and negative participants.

	Difference in rate of change in cortical thickness in %/year per 0.1 increment in baseline global Aβ SUVR (95% CI)	
	**ADsig Harvard**	**ADsig Mayo**
**Aβ positive (n=56)**	0.03 (-0.14, 0.20)	-0.17 (-0.36, 0.02)
**Aβ negative (n=281)**	-0.00 (-0.16, 0.15)	0.06 (-0.11, 0.22)

Coefficients and 95% confidence intervals are from a piecemeal linear spline regression model (with a knot value set to the SUVR cut point for Aβ positivity, 0.6104) adjusted for sex and age at baseline scan. Aβ = β-amyloid; SUVR = standardised uptake value ratio. ADsig Harvard consisted of entorhinal, inferior temporal, parahippocampal, temporal pole, precuneus, supramarginal, superior and inferior parietal, superior frontal, pars opercularis, pars triangularis and pars orbitalis areas.[1] ADsig Mayo was comprised of middle temporal, inferior temporal, entorhinal and fusiform areas.[2]

References

1. Dickerson BC, Bakkour A, Salat DH, et al. The cortical signature of Alzheimer's disease: Regionally specific cortical thinning relates to symptom severity in very mild to mild AD dementia and is detectable in asymptomatic amyloid-positive individuals. *Cereb Cortex*. 2009;19(3):497-510. doi:10.1093/cercor/bhn113
2. Jack CR, Wiste HJ, Weigand SD, et al. Different definitions of neurodegeneration produce similar amyloid/neurodegeneration biomarker group findings. *Brain*. 2015;138(12):3747-3759. doi:10.1093/brain/awv283
